# Compound heterozygosity of predicted loss-of-function *DES* variants in a family with recessive desminopathy

**DOI:** 10.1186/1471-2350-14-68

**Published:** 2013-07-02

**Authors:** Heather M McLaughlin, Melissa A Kelly, Pamela P Hawley, Basil T Darras, Birgit Funke, Jonathan Picker

**Affiliations:** 1 Laboratory for Molecular Medicine, 65 Landsdowne Street, Cambridge, MA, 02139, USA; 2 Laboratory for Molecular Medicine, Partners Center for Personalized Genetic Medicine, 65 Lansdowne St. Cambridge, Cambridge, MA, USA; 3 Department of Pediatrics, Division of Genetics and Metabolism, Boston Children’s Hospital Boston, 300 Longwood Avenue, Division of Genetics, Hunnewell 5, Boston, MA, USA; 4 Division of Clinical Neurology, Children’s Hospital Boston, Boston, MA, USA; 5 Department of Neurology, Harvard Medical School, Boston, MA, USA; 6 Department of Pathology, Massachusetts General Hospital, Boston, MA, USA; 7 Department of Pediatrics, Harvard Medical School, Boston, MA, USA

**Keywords:** Desminopathy, Myopathy, Dilated cardiomyopathy, Clinical genetics, Genetic testing

## Abstract

**Background:**

Variants in the desmin gene (*DES)* are associated with desminopathy; a myofibrillar myopathy mainly characterized by muscle weakness, conduction block, and dilated cardiomyopathy. To date, only ~50 disease-associated variants have been described, and the majority of these lead to dominant-negative effects. However, the complete genotypic spectrum of desminopathy is not well established.

**Case presentation:**

Next-generation sequencing was performed on 51 cardiac disease genes in a proband with profound skeletal myopathy, dilated cardiomyopathy, and respiratory dysfunction. Our analyses revealed compound heterozygous *DES* variants, both of which are predicted to lead to a loss-of-function. Consistent with recessive inheritance, each variant was identified in an unaffected parent.

**Conclusions:**

This case report serves to broaden the variant spectrum of desminopathies and provides insight into the molecular mechanisms of desminopathy, supporting distinct dominant-negative and loss-of-function etiologies.

## Background

The desmin gene (*DES*) encodes an intermediate filament protein important for maintaining cytoskeletal architecture in skeletal, smooth, and cardiac muscles [1]. Desminopathies are myofibrillar myopathies mainly characterized by progressive muscle weakness, conduction block, dilated cardiomyopathy, and in severe cases, sudden death. The overwhelming majority of disease-associated *DES* variants described thus far are missense variants inherited in a dominant fashion. These variants commonly result in a dominant-negative effect on wild-type proteins, leading to destabilization of desmin intermediate filament networks, accumulation of mutant desmin aggregates, and ultimately, myofibrillar disorganization and toxicity [2]. Only ~50 disease associated DES variants have been reported to date; and thus, the genotypic spectrum of desminopathy is not well characterized. In particular, the role of loss-of-function variants is not well understood.

## Case presentation

**Figure 1:**
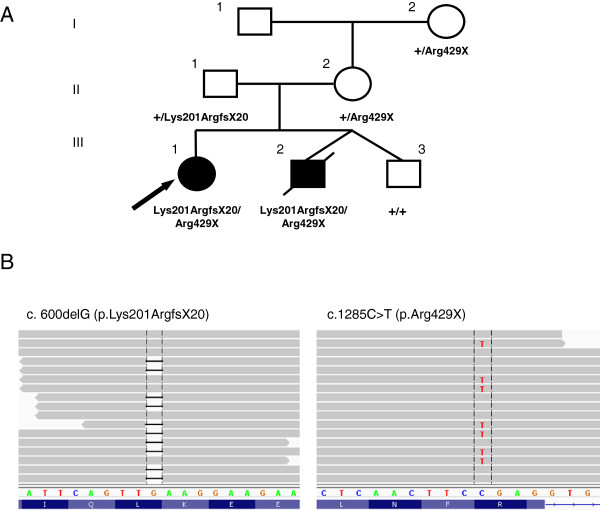
**Segregation and characterization of *DES *variants. (A)** Pedigree showing segregation of the Lys201ArgfsX20 and Arg429X variants. Filled symbols indicate individuals affected with desminopathy. Arrow indicates proband; + indicates wild-type *DES* allele. **(B)** Next generation sequencing alignments. Grey bars indicate no change from the reference sequence. *DES* DNA sequence is depicted by colored nucleotides, while the protein sequence is depicted underneath in blue. Vertical black lines indicate the nucleotide position where each variant resides.

The proband (Figure 1A; III-1, arrow), was the full term product of a pregnancy complicated by a reportedly prolonged period in the birth canal. Following vaginal delivery, she was noted to have a hemorrhage in the back of the brain which affected swallowing. Initially, she was also noted to be cyanotic (lips), hypotonic and lethargic. Though most of these issues resolved, hypotonia continued throughout childhood. Developmentally, she was essentially on schedule for gross, fine motor skills, and language; however, in addition to ongoing hypotonia, motor progress slowed in early childhood, with toe walking and stair climbing reported as concerns. The patient received Botox injections for tight heel cords at age 14 and reportedly became non-ambulatory shortly thereafter.

She was first evaluated clinically at our institution in the Neuromuscular Program at age 16, at which time she was alert and engaging with slightly dysarthric speech. She had mild ptosis and significant limitation of extraocular motility. There was no upgaze, however, there was some degree of downgaze, and also restricted lateral gaze. Her pupils were equal and reactive to light, and funduscopic examination showed flat discs with healthy optic nerve heads and no evidence of gross retinopathy. There was weakness of eye closure, and some degree of lower facial weakness. Her tongue was normal in volume without fasciculations and a gag reflex could be elicited.

Muscle strength studies revealed shoulder adduction was 4+/5, biceps and triceps were 4/5, wrist flexion and extension were 4+/5, hand grip was 4+/5, hip extension was 3+/5, hip abduction was 4-/5, hip extension was 2/5, knee extension was 5/5, tibialis anterior was 4+/5 on the right and 5-/5 on the left, tibialis posterior was 5/5, gastrocnemii were 5/5, peroneals were 4+/5, and her reflexes were 1-2+/5 and symmetric. In the sitting position, there was some degree of right-sided thoracic scoliosis. She was unable to walk without assistance and had a tendency to fall. Her finger:nose testing was normal and there was no evidence of truncal ataxia in the sitting position.

During her last visit in the Neuromuscular Program at age 27, she had marked dextroscoliosis, and contractures on her ankles, elbows, and hands bilaterally. She was tilted forward to facilitate the use of her wheelchair. She had very limited mobility in her lower extremities with dorsiflexion of 2/5 and 1/5 on the left and right foot, respectively. Her quads were 1/5 bilaterally. She had 3/5 wrist flexion on the right and 2+/5 on the left. She has no proximal movements in her arms and her shoulder shrug was 3/5. She has marked wasting of her muscles and winging of the scapula. She had very limited extraocular movements horizontally in both directions as well as in upgaze and downgaze and she had ptosis bilaterally. Her sensory exam was grossly intact.

The patient was also diagnosed with dilated cardiomyopathy at age 16. The cardiomyopathy is currently stable, but ventricular arrhythmias are increasing. She required placement of a tracheostomy to assist with breathing at age 22, and occasionally requires feeding assistance through a G-tube. There continue to be no cognitive concerns.

Diagnostic genetic testing for this patient has included chromosomal microarray analysis, POLG1 and RRM2B sequencing, mitochondrial genome sequencing, and congenital disorders of glycosylation testing, all of which were normal. The patient was first seen by the Genetic clinic at age 26 in an effort to establish an underlying genetic etiology for her presentation, where sequencing of 51 cardiomyopathy genes was ordered. The results from this test are summarized in the Results section.

Notably, the proband’s younger brother (Figure 1A; III-2), deceased at the time the proband was first evaluated by Genetics, was reported by parents to have had similar symptoms in infancy and in early childhood and was also reported to have had “floppy eyelids”. At the age of 13, this brother had a muscle biopsy of the right vastuslateralis, which revealed severe chronic myopathy with marked endomysial fibrosis and enlarged mitochondria with a complex pattern of cristae. He was also noted to have dilated cardiomyopathy at age 14. He died at 19 years of age, after developing a respiratory infection. Given the overlapping symptomatology, the parents elected to decline a muscle biopsy in the proband. The parents are currently asymptomatic; both are in their 50′s, and report having had normal echocardiograms within the last 5 years.

## Methods

SureSelect oligonucleotide-based target enrichment (Agilent Technologies, Santa Clara, California, USA) followed by next generation sequencing on the Illumina HiSeq2000 platform was used to sequence the coding regions and splice sites of 51 cardiomyopathy genes (for a list of genes see: http://pcpgm.partners.org/lmm/tests/cardiomyopathy). Variant calls were generated using the Burrows-Wheeler Aligner (BWA) followed by GATK analysis. Sanger sequencing was used to confirm variants and to perform familial sequencing. Variants are reported according to HGVS nomenclature (http://www.hgvs.org/mutnomen) using NCBI Reference Sequence NM_001927.3.

## Results

Sequence analysis of 51 cardiomyopathy genes revealed two heterozygous variants in the *DES* gene: c.600delG which is predicted to result in a frameshift and subsequent premature termination codon (p. Lys201ArgfsX20), and c.1285C > T which is predicted to result in a premature termination codon (p. Arg429X) (Figure 1B). Familial sequencing revealed both variants in DNA obtained from muscle tissue from the deceased sibling. The Lys201ArgfsX20 variant was inherited from the father and the Arg429X variant was present in the mother and maternal grandmother, all of whom are clinically unaffected at present (Figure 1A). These data establish the variants are present in trans (residing on separate chromosomes) in the proband and affected sibling, and support a recessive mode of inheritance in this family.

The Lys201ArgfsX20 variant in *DES* has not been previously reported in the literature or in population control databases, while the Arg429X variant in *DES* has been detected in 1/8600 European American chromosomes by the NHLBI Exome Sequencing Project (http://evs.gs.washington.edu/EVS/; dbSNP rs150974575). However, this does not conflict with a pathogenic role, as this individual may be presymptomatic (age and clinical information is unavailable for this cohort). Both variants lead to premature termination codons and are predicted to result in truncated or absent proteins. Based on the presentation in this family, these variants are likely to be pathogenic when present in combination, and unlikely to cause disease in isolation. Although the segregation of *DES* variants in this family supports a recessive inheritance, dominant effects with reduced penetrance or a milder presentation leading to late-onset disease cannot be ruled out.

## Discussion

The majority of desminopathies described to date are dominant; however, three cases exhibiting a recessive mode of inheritance have been reported including compound heterozygosity for two missense variants (p. Ala360Pro and p. Asn393Ile) in a family with severe, childhood-onset desminopathy, homozygosity for an in-frame deletion (p. Arg173_Glu179del) in a family with early-onset desminopathy and congenital sensory deafness, and recently, compound heterozygosity for loss-of-function variants (p. Thr76fsX21 and p. Glu108X) in a family with early-onset proximal muscle fatigue and weakness [3-5]. The family presented here features an early-onset desminopathy in siblings compound heterozygous for Lys201ArgfsX20 and Arg429X *DES* variants. Together, these data suggest that recessive desminopathies may lead to a severe, early-onset form of disease.

Nine predicted loss-of-function *DES* variants have been previously reported in the literature [5-11]. It is often assumed that loss-of-function variants lead to reduced or absent protein production due to nonsense-mediated decay (NMD) if the termination codon is introduced at least 50–55 nucleotides upstream of the 3′ end of the penultimate exon [12]. However, there are clear instances where loss-of-function variants fulfilling these criteria manage to escape NMD and produce a viable protein [13]. Functional studies indicate that some disease-associated loss-of-function *DES* variants result in impaired intermediate filament formation and aberrant desmin aggregation, suggesting that these variants do not result in a complete loss-of-function and instead lead to mRNAs that escape NMD and are translated in heterozygous individuals, resulting in a dominant-negative effect on the wild-type protein [6,7].

The three heterozygous family members of our index case carry predicted loss-of-function variants yet are currently unaffected, suggesting that these variants may lead to genuine loss-of-function, as the presence of truncated protein would be expected to lead to a phenotype in heterozygous individuals. It is predicted then, that the affected individuals in this family lack any functional desmin protein. While we were unable to analyze a muscle biopsy from our current case for desmin staining, a recent report of an additional family with recessive desminopathy caused by loss-of-function DES variants revealed a complete lack of desmin staining in a muscle biopsy from an affected individual in that family [5]. Interestingly, homozygous null desmin mice have been shown to develop cardiomyocyte hypertrophy and dilated cardiomyopathy characterized by extensive myocyte cell death, calcific fibrosis, and multiple ultrastructural defects [14,15]. Together, these data suggest that desminopathies may occur via two distinct molecular mechanisms: dominant-negative effects exerted by mutant desmin proteins and complete loss of the desmin protein. These mechanisms are expected to result in disorganization (in the case of dominant-negative effects) or absence (in the case off loss-of-function effects) of the desmin intermediate filament network and subsequent myofibrillar disorganization.

## Conclusions

We present compound heterozygous, predicted loss-of-function *DES* variants in two siblings with desminopathy. This study broadens the variant spectrum of desminopathy and provides evidence that both dominant-negative and loss-of-function effects play a role in the molecular pathogenesis of desminopathy.

## Consent

The patient has read and approved the manuscript and written informed consent was obtained.

## Competing interests

The authors declare that they have no competing interests.

## Authors’ contributions

HMM performed variant analysis and interpretation and drafted the manuscript. MAK performed variant analysis and interpretation and edited the manuscript. PPH provided genetic counseling to the family and assisted in clinical evaluation of the proband. BTD performed the neurological examination of the proband. BF performed variant analysis and interpretation and edited the manuscript. JP performed clinical evaluation of the proband and assisted in drafting of the manuscript. All authors have read and approved the manuscript.

## Pre-publication history

The pre-publication history for this paper can be accessed here:

http://www.biomedcentral.com/1471-2350/14/68/prepub
